# The Transactions Texas State Medical Association

**Published:** 1898-03

**Authors:** 


THE TRANSACTIONS TEXAS STATE MEDICAL ASSOCIATION.—In consequence of the epidemic of dengue in Galveston last summer, Secretary West was not able to get out the annual volume of transactions on time. It is usually issued about September, or October 1st. It was printed this year, as last, by the Von Boeckmann Publishing Co., of Austin, on the lowest and best bid. It was issued in February, ult. The JOURNAL acknowledges the receipt of a copy, but in consequence of a press of other business we have not as yet been able to examine it. We will endeavor to review it in our April number, which, by the bye, we will try to mail out on time, April 1st. The February number was delayed by the non-arrival of paper, and was two weeks late. We ask the indulgence of our patrons, as accidents will happen in the best regulated printing offices.

